# Classifying three imaginary states of the same upper extremity using time-domain features

**DOI:** 10.1371/journal.pone.0174161

**Published:** 2017-03-30

**Authors:** Mojgan Tavakolan, Zack Frehlick, Xinyi Yong, Carlo Menon

**Affiliations:** Menrva Research Group, Schools of Mechatronic Systems Engineering and Engineering Science, Simon Fraser University, Burnaby, British Columbia, Canada; University of Minnesota, UNITED STATES

## Abstract

Brain-computer interface (BCI) allows collaboration between humans and machines. It translates the electrical activity of the brain to understandable commands to operate a machine or a device. In this study, we propose a method to improve the accuracy of a 3-class BCI using electroencephalographic (EEG) signals. This BCI discriminates rest against imaginary grasps and elbow movements of the same limb. This classification task is challenging because imaginary movements within the same limb have close spatial representations on the motor cortex area. The proposed method extracts time-domain features and classifies them using a support vector machine (SVM) with a radial basis kernel function (RBF). An average accuracy of 74.2% was obtained when using the proposed method on a dataset collected, prior to this study, from 12 healthy individuals. This accuracy was higher than that obtained when other widely used methods, such as common spatial patterns (CSP), filter bank CSP (FBCSP), and band power methods, were used on the same dataset. These results are encouraging and the proposed method could potentially be used in future applications including BCI-driven robotic devices, such as a portable exoskeleton for the arm, to assist individuals with impaired upper extremity functions in performing daily tasks.

## Introduction

Brain-computer interfaces (BCIs) are a promising tool for detecting user intention and controlling robotic devices [1, 2]. While both invasive and non-invasive methods have been proposed for acquiring brain signals [3, 4], this study focuses on a non-invasive BCI based on EEG. BCIs rely on signal processing to identify changes in brain activity corresponding to different mental tasks. Because EEG signals have a very low signal-to-noise ratio [5], signal processing is particularly important and generally involves two related steps: feature extraction and classification [6, 7]. The selection of features has a direct impact in determining the performance of a classifier—choosing inappropriate features may lead to poor classification performance [8].

Various combinations of extracted features and classification algorithms, with different degrees of complexity and efficiency, have been proposed in the literature for EEG signal processing [9, 10]. Widely used feature extraction methods include common spatial patterns (CSP) [11], filter bank CSP (FBCSP) [12, 13], band power [5], mean absolute value (MAV), zero crossings, and band slope sign changes [14]. Classification algorithms that are common in BCI research include support vector machine (SVM) and linear discriminant analysis (LDA) [15]. Different feature and classifier combinations may be preferable depending on the BCI control tasks and system design [15].

Time-domain features have low computational complexity and could be an appropriate option for real-time BCI systems [14]. Time-domain features such as Willison amplitude (WAMP), simple square integral (SSI), MAV, maximum absolute value (MAX), and waveform length were evaluated in a study that classified four motor imagery tasks (right hand, left hand, tongue on the right and left side of the mouth) using SVM and Fuzzy C-means (FCM) [16]. A classification accuracy of 85.07% was achieved with WAMP features and an SVM classifier [16]. Other studies explored the use of time-domain approaches such as adaptive autoregressive modelling [17–19]. While these preliminary studies reported that time-domain features can be promising, further research is needed to investigate their potential use in a variety of different motor imagery tasks.

Most existing BCI systems can only recognize a limited number of mental tasks as control commands. Left hand, right hand, and foot motor imagery tasks are among the most frequently used for controlling BCI systems [6]. It is known that detecting the intention or the motor imagery of different movements within the same limb is particularly challenging. This is due to the fact that same limb motor tasks activate regions that have close representations on the motor cortex area of the brain [20, 21]. There have been relatively few studies that address this challenge.

Several studies investigated the decoding of different wrist movements [22, 23]. Vuckovic and Sepulveda used Gabor transform (time-frequency) features and an Elman neural network to classify four wrist movements (flexion, extension, pronation, and supination) with an average accuracy of 71.3% for each pair [24]. In a later study using the same technique, Vuckovic and Sepulveda reported an average accuracy of 67.5% for classifying flexion against extension [25]. Most recently, Edelman *et al*. reported that source space analysis can improve the classification accuracy of wrist movements [26]. Using band power features to classify four wrist movements, an average accuracy of 69.1% was achieved for traditional sensor space EEG and 81.4% for source space analysis. Another study used band power and movement related cortical potential (MRCP) features to classify two wrist movements (extension and rotation) and two movement speeds (fast and slow) [27]. That study reported an average accuracy of 79% for movements of different speeds, but did not report classification accuracy for movements with the same speed.

Meanwhile, a few studies investigated non-wrist movements within the same limb. Liao *et al*. classified ten different pairs of voluntary finger movements (i.e. index vs. middle, thumb vs. ring, etc.) with an average accuracy of 77.1%, using power spectral changes as features and an SVM classifier [28]. The high classification accuracy in that study can be attributed to use of a high density 128 electrode array. Lopez-Larraz *et al*. classified movement intention for seven different upper arm joints with an average accuracy of 57.9% [29]. It should be noted that the objective of that study was limited to detecting the onset of a movement versus rest.

Generally, the difficulty of classification can be explained by the fact that all joints of a single limb are closely represented on the motor cortex [20, 21] and that EEG signals have non-stationary characteristics [30]. To tackle this challenging classification problem, the most common approaches proposed in the literature for classifying same limb motor imagery tasks were based on band power or frequency-based features. Additional processing techniques, such as independent component analysis (ICA), were also used to improve classification [22], with source space analysis being a more recent development. It should be noted that nearly all studies used EEG systems with at least 64 electrodes.

Our previous study (PS) [15] investigated the feasibility of classifying rest against two imaginary movements within the same limb (elbow and hand), using several widely-used state-of-the-art methods. Specifically, nine different schemes were tested: the combination of three different feature extraction methods (CSP [11], FBCSP [12, 13], and band power [5]) and three different classification methods. To maximize accuracy, classification training was performed individually for each of the twelve subjects. PS demonstrated an average classification 3-class accuracy of 58.4%, whereas the 2-class accuracy for classifying elbow against grasp was 63.9%. Considering that PS used a reduced number of electrodes (i.e. 32 channels), the results were deemed to be consistent with the literature. The relatively low accuracy that was obtained however motivated the authors to investigate potential new approaches.

In this work, we propose the use of time-domain features to tackle the problem of classifying imaginary movements within the same limb. The dataset from PS was used to fairly and objectively compare the performance of this novel method against well-established methods proposed in the literature. The tackled problem was to discriminate rest, imaginary grasp, and imaginary elbow movements performed with the same limb, specifically the right arm. We considered this to be a 3-class problem, although the 2-class comparison of elbow and grasp conditions was expected to be more difficult, as highlighted in our previous study [15]. The rest case was included because it made the BCI more appropriate for real-world use. Motor imagery of grasp and elbow movements were chosen due to their potential for intuitively controlling the robotic arm [31] developed in our lab. For example, the user could imagine elbow movements to move the robotic device close to a cup, and then imagine grasping movements to grab the cup. The proposed BCI extracted features from the EEG signals using time domain features including autoregressive (AR) model coefficients, root mean square (RMS), and waveform length (WL). The features were classified using a multiclass support vector machine (SVM) with optimized parameters. Our results were then compared with those from PS where well-known methods such as CSP [11], FBCSP [12, 13], and band power [5] were used. This comparison enabled a straightforward evaluation of the usefulness of our proposed features relative to the state-of-the-art.

## Experimental procedure

### Data collection

This work used the data set previously described in PS [15]. All of the methods within this study were in compliance with the declaration of Helsinki and were approved by the Office of Research Ethics (#2012s0527), Simon Fraser University (SFU). Twelve able-bodied individuals were recruited, each of whom gave written informed consent before participating in the experiment.

**Fig 1:**
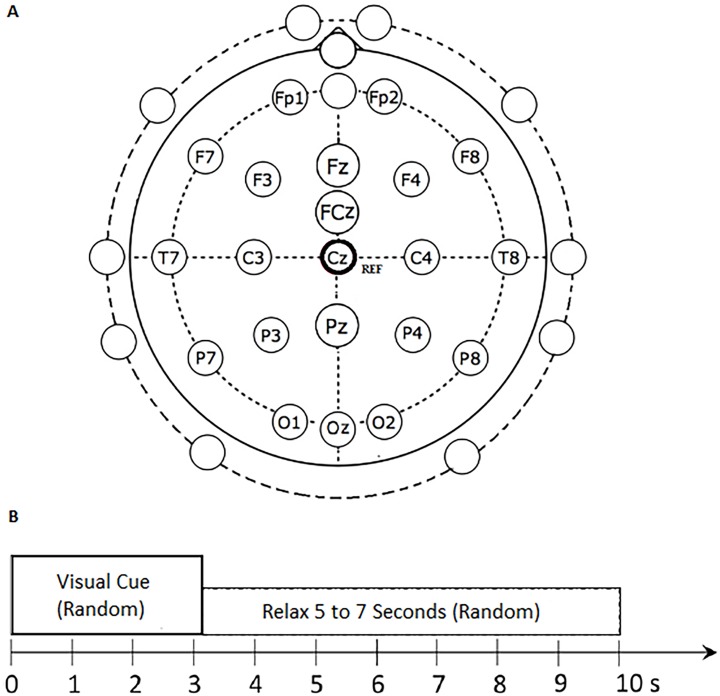
The EEG electrode positions employed in this study [15].

The experiment recorded non-invasive EEG data using a 32-channel EGI Geodesic Sensor Net (Electrical Geodesics, Inc., Eugene, OR, USA) [32]. The EEG signals were amplified and sampled at 1000 Hz using an EGI Geodesic Net Amps 400 series amplifier [33]. The data were then transmitted via the TCP/IP protocol to a computer. Throughout the experiment, the electrode impedance was maintained below 50 kΩ. Fig 1 shows the locations of all the electrodes of the sensor net [15]. The labelled electrodes are those that were used in our proposed method. The unlabelled electrodes, on the other hand, were not considered because they were very close to muscle EMG sources and therefore most susceptible to artifacts [15]. The vertex (Cz) was selected as the reference electrode because it is commonly used in literature [34] and often adopted by EEG recording equipment, including the EGI system we used [35, 36].

During data collection, the participants were seated comfortably in front of a computer monitor which displayed visual cues. Three different cues were presented, each corresponding to a designated task: REST (rest and relax); MI-GRASP (imagine opening and closing all the fingers to grab an object); and MI-ELBOW (imagine moving the forearm up and down) [15]. The experiment lasted for approximately 1.5 hours for each participant, consisting of four 12 minute sessions. Each session consisted of 20 trials for each task, with each trial lasting for 8 to 10 seconds. Fig 1(b) illustrates the trial structure. First, a random visual cue was displayed for 3 seconds, during which the participant performed the designated motor imagery task, followed by a 5 to 7 second rest interval. Throughout the experiment, the participant took a break whenever needed.

### EEG data pre-processing

Following PS [15], the EEG data were down-sampled to 250 Hz and then band-pass filtered in the frequency band 6–35 Hz. This frequency range encompasses both the mu and beta rhythms which desynchronizes during motor imagery [37], which has been successfully used in BCI systems for classifying EEG signals [11, 38–39]. Also, band-pass filtering minimizes the effects of ocular artifacts resulting from eye movement and blinks, which contaminate mainly the low frequency components of the EEG data.

### Proposed method

#### Feature extraction

Feature extraction highlights important information and eliminates redundant or non-informative data. EEG signals are transformed into a feature vector, which is then classified by a machine learning algorithm. In this study, three types of time-domain features were computed based on signal amplitudes: AR model coefficients, WL, and RMS. These features require no complex calculation [40].

The first set of features was obtained using AR modeling. AR models the data such that the current sample is a weighted linear combination of its previous samples and thus provides information regarding the previous samples [41]. The mathematical representation of AR model coefficients is expressed by Eq (1):

$$t_{n}=\overset{p}{\underset{i=1}{\sum }}a_{i}t_{n-i}$$ (1)

where {*a* for *i* = 1,…, *p*} are AR model coefficients and *p* is the model order.

Various AR models were evaluated on a fairly large database of EEG segments by [42] for EEG feature extraction. Using inverse filtering and one-second EEG segments, they found that AR models of orders between 2 and 32 yielded the best EEG estimation. For this study, the coefficients of fourth-order AR models were used to represent the EEG signals [41].

The second feature set is WL, which provided a measure of the waveform complexity in each segment. It can be mathematically represented by Eq (2):

$$y=\overset{N}{\underset{i=1}{\sum }}\left|\Delta w_{i}\right|=\overset{N}{\underset{i=1}{\sum }}\left|w_{i}-w_{i-1}\right|$$ (2)

where *w*_*i*_ is the amplitude of the *i*^th^ sample and *N* is the number of samples.

The third extracted feature set is RMS, which has been used in processing of EEG signals [40, 43]. RMS provides information related to the amplitudes of the EEG signals. This feature is computed by Eq (3):

$$\text{RMS}=\sqrt{\frac{r_{1}^{2}+r_{2}^{2}+\ldots +r_{n}^{2}}{n}}$$ (3)

where *r*_*i*_ is the amplitude of the *i*^th^ sample and *n* is the number of samples.

For this study, the EEG signals were segmented using a window size of one second or 250 samples [14]. From each time segment and each EEG channel, a total of six features were extracted: four from AR, and one each from RMS and WL. Twenty channels of EEG data were used, so that a total of 120 features were generated and fed to the classifier.

#### Classification

Classification was performed using an SVM classifier, an efficient state-of-the-art method [44]. SVM finds discriminant hyper-planes and separates the data that belong to different classes with the maximum possible margin [45]. Maximizing the margins increases the generalization capabilities of the SVM classifier. In its general formulation, SVM requires solving the following optimization problem:

$$Min\;\frac{1}{2}{\left\|a\right\|}^{2}+c\overset{N}{\underset{i=1}{\sum }}\xi_{i}$$ (4)

$$subject\;to\;w_{i}y\left(x_{i}\right)\;\geq 1-\xi_{i}\;where\;i=1\;\ldots \;N\;and\;\xi_{i}\geq 0$$ (5)

where *y* is the learned model, *c* is the penalty factor, *a* is the vector representing adaptive model parameters, *w*_*i*_ is the label associated with a data point, *i* is the index associated with a data point, *ξ*_*i*_ is the slack variable, *x*_*i*_ is the vector representing a data point, and *N* is the number of data points.

A linear SVM can make non-linear decision boundaries by using the ‘kernel trick’ [45]. It is generally done by mapping the data to higher dimensionality space, with the help of a kernel function [45]. The radial basis function (RBF) was selected as the kernel function for this study. RBF has the smallest number of hyper-parameters compared to other kernel functions (such as the polynomial kernel [46]), so that RBF is less computationally expensive. The mathematical representation of the RBF kernel is:

$$k\left(y_{i},y_{j}\right)=exp\;\left(-\gamma {\left\|y_{i}-y_{j}\right\|}^{2}\right)$$ (6)

where *γ* is the kernel parameter and *y*_*i*_, *y*_*j*_ are training vectors.

The classification performance of the SVM depends on two parameters: 1) the kernel parameter gamma *γ*, which determines the convergence tolerance, and 2) the penalty weight *c*, which is a regularization parameter that controls the misclassification rate of the training data. The influence of these parameters was tested using a grid search, along with cross-validation, in the interval (0, 3) for *γ* and (0, 100) for *c*. The values of the parameters that resulted in the highest cross-validation accuracy were then selected to build the optimal SVM classifier.

To implement multiclass classification using SVM, we used a one-versus-one strategy. More specifically, classification was performed using a maximum wins voting strategy, in which every classifier assigns the data to one of two classes [47] and the assigned class receives one vote. The class that receives the highest number of votes is selected as the final class. Thus, considering *n* as the number of classes, this approach involves building *n* (*n*-1)/2 classifiers. In our case, *n* = 3 and for each 3-class classification problem, 3 binary classifiers were set up. In a three-class classification problem, the output of the classifier had one of the three discrete states ‘0’, ‘1’, or ‘2’ and was not a continuous function. The logical states ‘1’ and ‘2’ indicated the user’s intention to activate a device (e.g. a robotic arm). The logical state ‘0’, on the other hand, implied that the user did not intend to activate the system. To evaluate the performance of the 3-class BCI system, the data set was randomized and divided into ten folds [47]. Nine of the folds were used to set up the classifier and the remaining one fold was used to test the classifier [47]. The ratio of training data to testing data is consistent with literature showing that this ratio is appropriate and generally used for real-time EEG-based BCIs [6, 48–49]. Using this cross-validation procedure could help prevent over-fitting.

### CSP, FBCSP and band power methods

Several different approaches have been proposed to extract discriminative features of motor imagery from EEG signals, with varying degrees of success [50, 51]. CSP [11], FBCSP [12, 13], and band power [5] methods are among the most widely used feature extraction methods in BCI research. Thus, these three methods were employed in this study and their performances were compared with that achieved using the proposed time-domain features.

An open-source MATLAB toolbox, BCILAB, was used to process the acquired EEG data [52]. The EEG data were pre-processed as previously described, then divided into epochs from 1 to 3 s after the cue was given to the participant [15]. CSP [11], FBCSP [12, 13], and band power [5] methods were applied to these EEG epochs. For the FBCSP method, as in PS [15], the signal was broken down into 7–15 Hz, 15–25 Hz, and 25–30 Hz frequency sub-bands. For each EEG epoch, 6, 18, and 20 features were respectively obtained from CSP [11], FBCSP [12, 13], and band power [5] methods.

The features were then used to train a classifier using optimized SVM [15, 47] for each participant. To optimize multiclass classification using SVM, the grid search strategy along with cross-validation was applied for all the classification models [15, 47]. To evaluate the performance of the classifier, a 10 x 10 cross-validation method [15, 47] was employed. To achieve multiclass classification, the same one-versus-one strategy was applied to all the classification models [15, 47].

## Results

**Fig 2:**
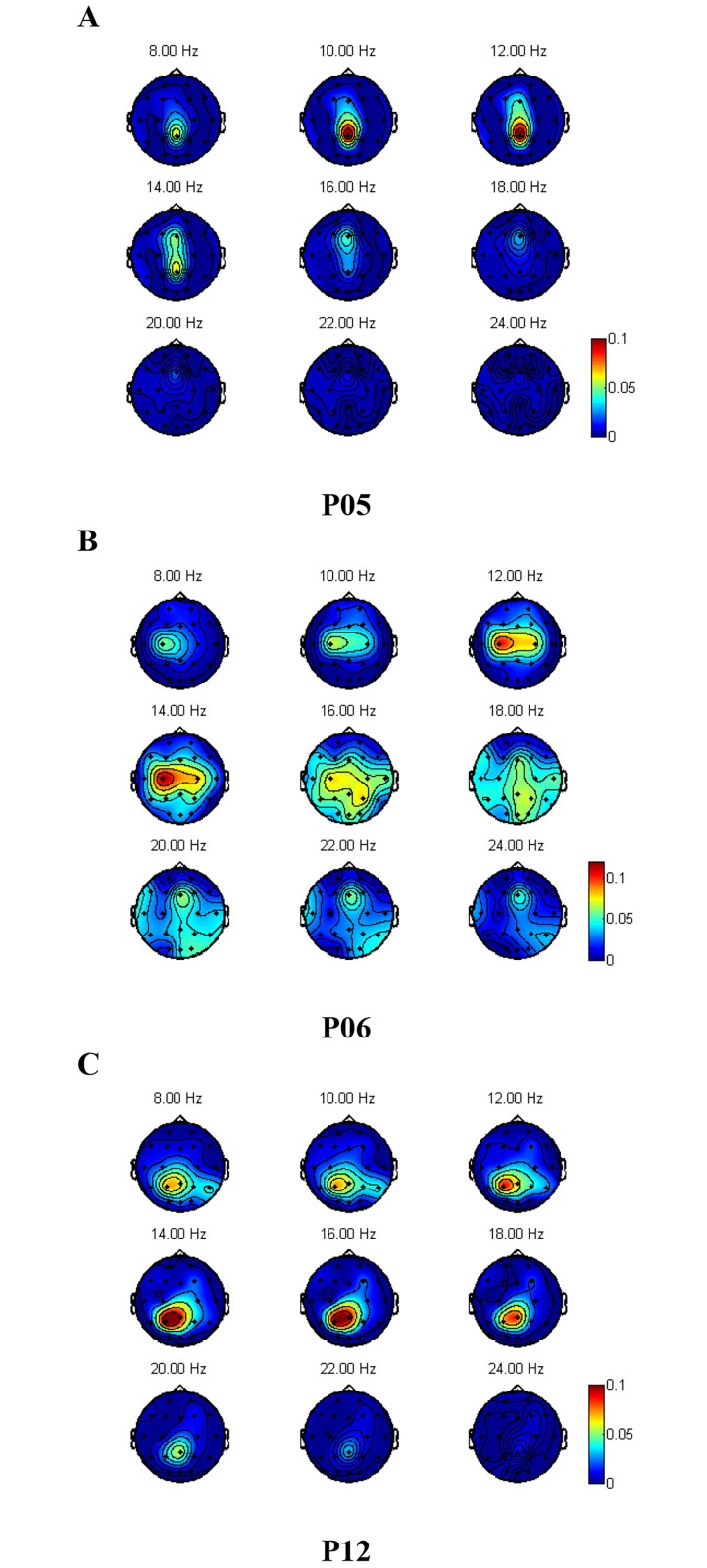
*R*^2^ values when different MI tasks were performed. (a) MI-GRASP vs. MI-ELBOW for participant P05. (b) MI-GRASP vs. MI-ELBOW for participant P06. (c) MI-GRASP vs. MI- ELBOW for participant P12.

Topographical analysis can highlight activity changes in different areas of the brain during different imaginary movement tasks. Topographical distributions of band power, for frequencies between 8–24 Hz, were generated for each participant and each motor imagery condition [15]. These distributions were then compared, in terms of *R*^2^ values, to identify the scalp areas that differentiate each pair of imaginary movements. For example, the topographical comparison of MI-GRASP and MI-ELBOW for participants P05, P06 and P12 is presented in Fig 2(a), 2(b) and 2(c) respectively. The observed topographical differences were participant-specific, so that no consistent patterns could be observed between participants, which is consistent with PS [15]. Such participant-specificity led the authors of this work to build subject-specific models for the classification.

For the case of MI-GRASP vs. MI-ELBOW, which involve activation of very close areas in the motor cortex (both detected by C3 of our 32-channel EEG cap), large R^2^ values were observed in different areas of the brain. This result may not be surprising given that imagined arm and hand movements generally result in multiple areas of the brain becoming excited [6, 53, 54]. Differences between subjects might be attributed to the subjective way the participants formulated their imaginary movements, thus requiring different brain areas to be activated.

Table 1 presents the maximum *R*^2^ values obtained using the power of the frequency bands ranging from 8–24 Hz [15] and the maximum *R*^2^ values obtained using the RMS, WL, and Autoregressive coefficients of our proposed method. The table lists the maximum MI-GRASP vs. MI-ELBOW *R*^2^ values (from any electrode) for each participant and the averaged value obtained for all the participants (P01-P12). For every participant, the *R*^2^ value for our proposed features is larger. This suggests that our time-domain features can improve the classification. Using the SVM classifier these features were evaluated and the obtained results from the selected and optimal classification models are presented in Table 2.

**Table 1:** Maximum *R*^2^ values for differentiating MI-GRASP and MI-ELBOW tasks. *R*^2^ values generated using the power of the frequency bands ranging from 8–24 Hz [15] are presented by F_*R*^2^. *R*^2^ values generated using RMS, WL, and Autoregressive model coefficients are presented by T_*R*^2^.

Participant	F_*R*^2^	T_*R*^2^
P01	0.03	0.42
P02	0.03	0.65
P03	0.12	0.73
P04	0.06	0.50
P05	0.08	0.50
P06	0.12	0.50
P07	0.10	0.67
P08	0.07	0.47
P09	0.04	0.58
P10	0.17	0.67
P11	0.02	0.44
P12	0.11	0.75
Mean	0.08	0.57

**Table 2:** Comparison of the performance obtained using different *γ* and *c* values.

Participant	Selected	*c*,10	*c*,20	*c*,30	*c*,40	*c*,50	*c*,60	*c*,70	*c*,80	*c*,90
	*c*,*γ*	*γ*,*0*.*2*	*γ*,*0*.*3*	*γ*,*0*.*5*	*γ*,*0*.*7*	*γ*,*0*.*9*	*γ*,*1*.*1*	*γ*,*1*.*3*	*γ*,*1*.*5*	*γ*,*2*.*5*
P01	76.8	59.6	65.5	70.7	72.9	73.4	73.9	75.2	75.3	76.7
P02	70.3	56.1	61.0	66.1	68.2	68.5	68.9	69.0	69.3	70.2
P03	77.2	61.7	66.7	71.6	73.9	75.2	75.9	76.3	76.6	76.9
P04	74.8	50.7	57.8	63.5	69.0	72.1	73.1	73.3	73.2	74.6
P05	73.2	52.9	56.7	58.9	62.1	65.0	67.4	69.5	71.2	73.0
P06	75.0	56.9	62.9	68.5	69.7	71.8	73.3	73.6	73.8	75.0
P07	71.9	55.8	63.0	67.9	69.9	69.6	69.9	70.6	70.9	71.6
P08	75.5	51.2	60.5	66.9	69.9	71.1	71.5	72.4	72.5	74.7
P09	72.2	55.0	61.1	68.6	69.6	70.3	71.7	71.4	71.7	72.1
P10	73.9	61.8	65.1	68.5	71.8	73.0	73.3	73.4	73.6	73.0
P11	75.7	48.9	54.8	61.1	67.8	69.6	70.7	72.7	73.4	75.5
P12	74.5	52.1	58.5	66.6	70.0	70.8	72.0	72.3	73.2	74.1
Mean	74.2	55.2	61.1	66.6	69.6	70.9	71.8	72.5	72.9	74

**Fig 3:**
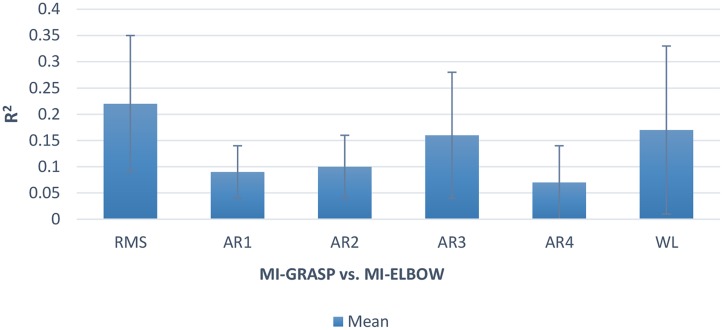
*R*^2^ values investigated for RMS, WL, and autoregressive coefficients over the sensorimotor cortex.

The maximum *R*^2^ values for RMS, WL, and Autoregressive coefficients were investigated specifically over the sensorimotor cortex. The maximum variation observed in RMS, Autoregressive coefficients and WL was respectively 0.5, 0.4 and 0.5 over the sensorimotor cortex (C3 and C4 electrodes). These results highlight that the motor cortex was highly activated across subjects during the motor tasks as expected. The average *R*^2^ values for RMS, WL, and Autoregressive coefficients were also investigated. The average of variation over the sensorimotor cortex (C3 and C4 electrodes) is presented in Fig 3. RMS (with the average *R*^2^ value of 0.22) provides information related to the amplitudes of EEG signals and WL (with the average *R*^2^ value of 0.17) is the cumulative length of the waveform over the segment. WL indicates a measure of waveform amplitude, frequency and duration, all within a single parameter [40]. Autoregressive coefficients (with the average *R*^2^ value of 0.1) provide information regarding the previous samples [41].

**Fig 4:**
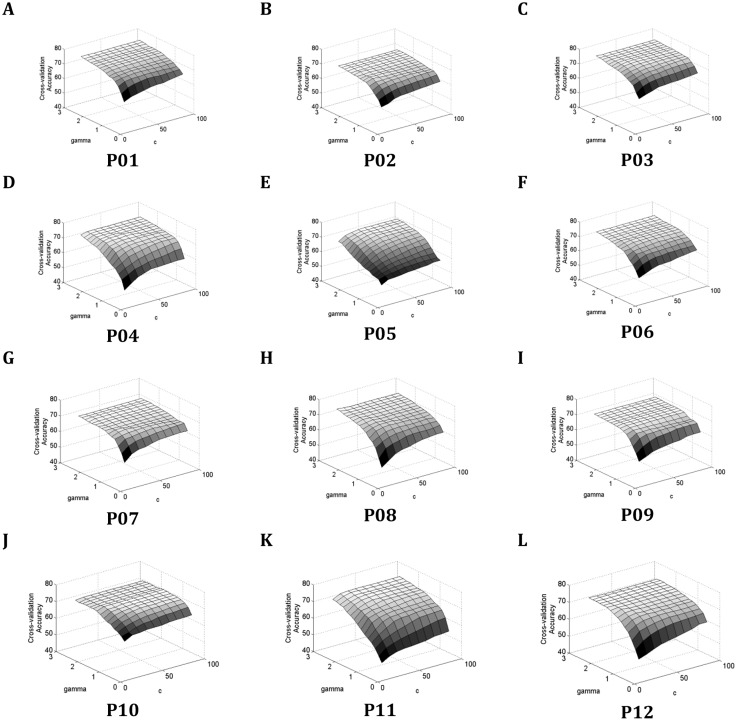
Cross validation accuracies of REST vs. MI-GRASP vs. MI-ELBOW based on *c* and *γ* parameters. (a) Participant P01. (b) Participant P02. (c) Participant P03. (d) Participant P04. (e) Participant P05. (f) Participant P06. (g) Participant P07. (h) Participant P08. (i) Participant P09. (j) Participant P10. (k) Participant P11. (l) Participant P12.

Fig 4 shows cross-validation accuracy for all participants when the parameters *c* and *γ* of the SVM classifier were changed. Table 2 presents the classification performance obtained using optimal parameters as well as other kernel parameters. An average accuracy of 74.2% was obtained when selecting the optimal parameters, which was 19% higher than when using parameters with the least performance (for *c* = 10 and *γ* = 0.2 the average accuracy dropped to 55.2%;). This result shows that performance is highly sensitive to these parameters and highlights the importance of selecting the optimal SVM parameters.

## Discussion

The optimal SVM parameters used in the proposed classification scheme and the average cross-validation accuracy achieved for each participant are presented in Table 3. The overall results obtained from the proposed method are encouraging as the obtained accuracy was greater than 70.0% for nine participants. The performance of the proposed method was compared to well-established methods presented in the literature which were investigated in [15] using the same dataset. The method proposed in this work (74.2 ± 2.1% average accuracy) outperformed by 18% the methods investigated in [15] (56.2 ± 8.5 average accuracy when optimal subject-specific methods, out of the nine investigated, were considered).

**Table 3:** The optimal *c* and *γ* values and comparison between the performance of the proposed method and PS [15].

			Proposed Method	PS [15]
Participant	Selected *c*	Selected *γ*	Accuracy (%)	Accuracy (%)
P01	10	2.5	73.7 ± 3.1	58.2 ± 9.6
P02	10	2.5	68.3 ± 2.6	62.8 ± 9.3
P03	10	2.4	74.8 ± 3.0	65.9 ± 8.9
P04	25	2.3	70.9 ± 4.7	53.1 ± 8.3
P05	40	2.5	66.5 ± 5.4	58.8 ± 9.0
P06	15	2.5	71.9 ± 3.5	61.1 ± 9.1
P07	25	2.1	70.0 ± 2.3	56.3 ± 8.8
P08	10	2.5	71.0 ± 4.1	47.6 ± 9.6
P09	15	2.3	70.1 ± 3.0	52.5 ± 9.7
P10	45	1.4	71.8 ± 2.4	70.3 ± 9.1
P11	25	2.4	69.6 ± 6.1	40.1 ± 9.7
P12	10	2.4	71.1 ± 4.2	48.3 ± 8.8

**Fig 5:**
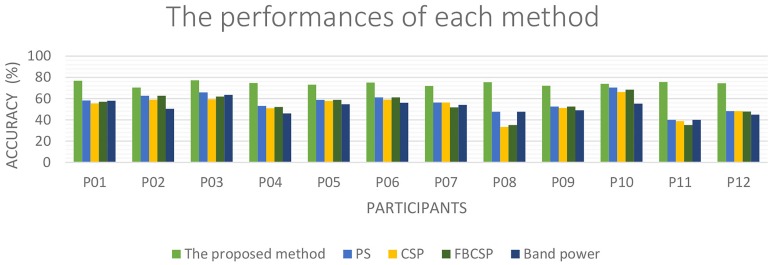
The classification accuracies of different methods. The classification accuracies of REST vs. MI-GRASP vs. MI-ELBOW by applying the proposed method, PS [15], CSP, FBCSP and band power methods for each individual.

**Fig 6:**
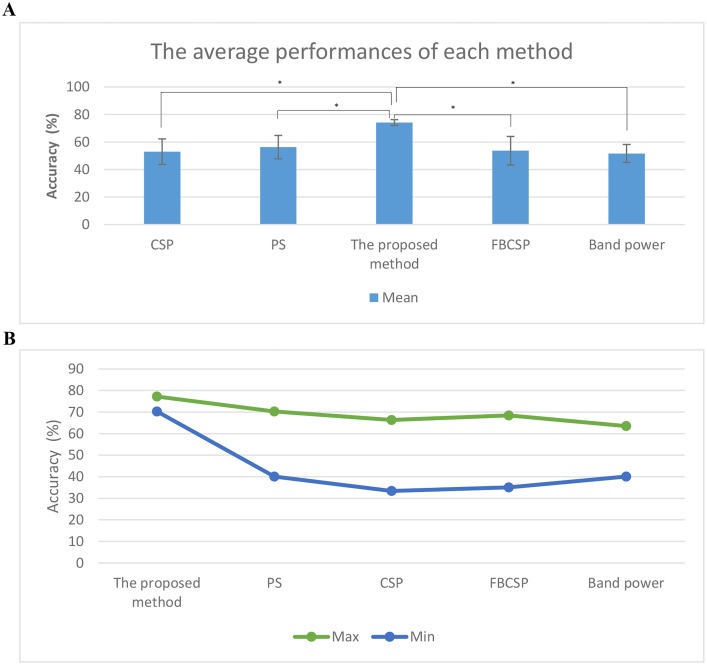
The average, maximum and minimum classification accuracies. (a) The average classification accuracies of REST vs. MI-GRASP vs. MI-ELBOW by applying the proposed method, PS [15], CSP, FBCSP and band power methods. (b) The minimum and maximum performance obtained by applying the proposed method, PS [15], CSP, FBCSP and band power methods.

Fig 5 compares the performance of the proposed method with that reported in PS [15] as well as that obtained when a SVM was used to classify CSP, FBCSP and band power features. This figure illustrates that the proposed method had the best performance for each individual participant. Meanwhile, the average classification accuracy for each method is presented in Fig 6(a). CSP, FBCSP and band power methods achieved an average classification accuracy of 53 ± 9.3%, 53.7 ± 10.4% and 51.7 ± 6.5% respectively for the classification of REST vs. MI-GRASP vs. MI-ELBOW. The repeated measure analysis of variance shows a statistically significant difference (*p*-value < 0.01) in mean accuracy scores between methods. The post hoc tests using the Tukey Kramer adjustment [55] shows a statistically significant difference in mean accuracy scores between the proposed method and CSP, FBCSP, Band power, and PS at the alpha 0.05 level of significance. The 95% confidence interval for the mean difference in accuracy scores between the proposed method and PS is (12.2, 23.8). The 95% confidence interval for the mean difference in accuracy scores between the proposed method and CSP is (15.5, 27.0). The 95% confidence interval for the mean difference in accuracy scores between the proposed method and FBCSP is (14.7, 26.3). The 95% confidence interval for the mean difference in accuracy scores between the proposed method and Band power is (16.8, 28.4).

Fig 6(b) presents the maximum and the minimum accuracy values among the individual participants for all methods. The maximum accuracy achieved was 77.2% (participant P03) using the proposed method whereas the lowest accuracy was 33.4% (participant P08) using the CSP method. The performance of the FBCSP method was very close to that of the CSP method (i.e., 35.2% for participant P08). This figure shows that the proposed method has much less inter-individual variability than the other methods. The lowest individual performance for the proposed method was equivalent to the highest individual performance in PS. This is a very strong attribute, as most studies in the literature have reported large performance variability between individuals. Our proposed method could potentially make BCI performance more reliable for all individuals.

**Fig 7:**
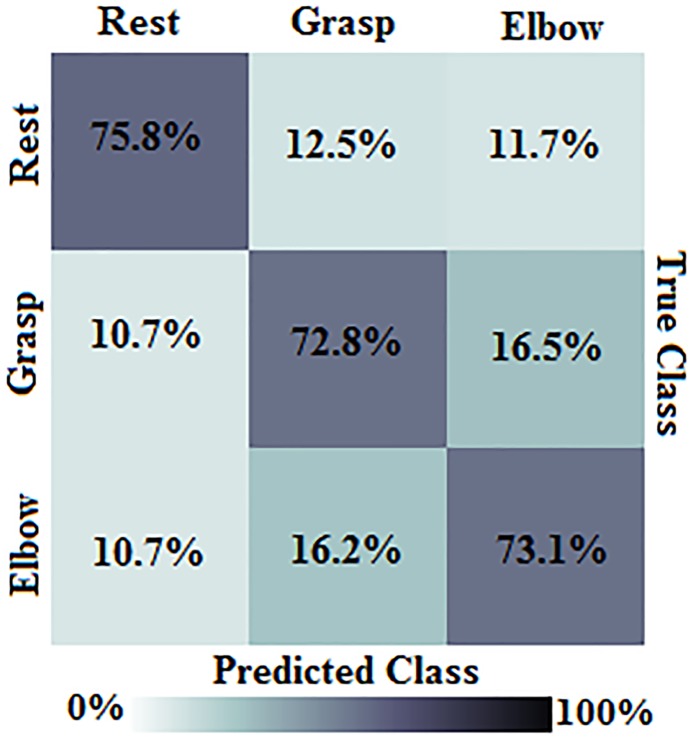
The three class confusion matrix. Group average rates of correct classification and misclassification for REST, MI-GRASP, and MI-REST tasks.

Fig 7 presents a confusion matrix showing group-level classification results for each motor imagery task. The average classification accuracy was 75.8% for rest, 72.8% for grasp, and 73.1% for elbow. Although REST has a slightly higher accuracy (which is reasonable considering the similarity of MI-GRASP and MI-ELBOW), the accuracies are encouragingly similar overall. However, the distribution of misclassification errors is notably different for each task. MI-GRASP is most frequently misclassified as MI-ELBOW (16.5%), and vice versa (16.2%). The movement tasks are each misclassified as REST only 10.7% of the time. Meanwhile, REST is misclassified as MI-GRASP and MI-REST at similar rates (12.5% and 11.7%, respectively). The fact that MI-GRASP and MI-ELBOW are more likely to be misclassified as each other agrees with our initial expectation that this was the most difficult two-class problem. This imbalance of classification errors could negatively impact BCI usability. Misclassification between movement tasks would cause unintended movement of the robotic device, which may be more undesirable than a simple failure to initiate a movement. This consideration could be addressed in future work.

It should be noted that while PS used popular feature extraction methods for motor imagery EEG classification [6, 13, 51], it reported lower performance than some other studies using same limb tasks. In fact, PS reported a two-class accuracy of 63.9% [15], whereas a typical study in the literature reported 71.2% for classifying wrist movements [24]. Such a lower accuracy, as briefly mentioned in the introduction, can be attributed to the fact that only 32 electrodes were used in PS whereas in most of the other studies at least 64 electrodes were used. Despite this fact, this new study demonstrated an accuracy of 74.2% using the same 32 electrode device and dataset reported in PS. The performance of our proposed method therefore already compares favourably with the literature and could improve further with the use of a higher density EEG device.

The use of a higher density EEG device could also decrease the topographical differences between subjects. For the case of MI-GRASP vs. MI-ELBOW, high variability was shown between subjects (see Fig 2), which was consistent with PS [15]. The imagined arm and hand movements resulted in excitation of multiple areas of the brain [6, 53, 54]. A higher electrode density might better reveal shared patterns between subjects. These tasks also require the activation of very similar areas in the motor cortex, which are difficult to discriminate using a low density EEG device. Having more electrodes in the sensorimotor area would help make this problem less severe. The variability among subjects could also be attributed to the fact that individuals imagine movements in slightly different ways.

The time-domain features (WL, RMS, and AR coefficients) proposed here have several other advantages. They are not computationally intensive, making them easy to implement in a real-time BCI application. In this paper, these time-domain features are calculated on epochs of one second. Meanwhile, band power and time-frequency features in literature are typically derived from 2–3 second epochs [25, 27]. Therefore, a BCI based on our proposed features could have a faster response time and higher information transfer rate (ITR). On the other hand, one problem with the current method is that it requires approximately 1 hour of training data from each user. This is a barrier to use that should be addressed in future work.

It should be noted that this study intentionally did not implement a source space analysis, which was shown in [26] to boost an initial accuracy of 69.1% to 81.4%. This type of analysis was excluded in order to narrow the focus of this work, whose primary objective was to investigate the potential benefits of using time-domain features. The authors believed that a direct comparison with PS, which used the same dataset and implemented state of the art features, was best suited to provide evidence that time-domain features could be considered to improve classification accuracy. Future works should however investigate the possibility of combining the findings of this work with those presented in [26] and other studies.

## Conclusions

In this study, we proposed a scheme for processing EEG signals and discriminating among three different motor imagery tasks all involving the same limb (i.e. rest, imaginary grasp, and elbow movements). This scheme involves the use of time-domain features along with an optimized SVM with an RBF kernel. The time-domain features extracted include the AR model coefficients, RMS, and waveform length.

The use of SVM classification was shown to be suitable for discriminating rest against two imagined arm movements from the same limb. It was demonstrated that selecting suitable EEG features and optimizing the SVM parameters are key to obtaining good classification performance. Our results show that successful classification of imaginary movements within the same limb can be achieved. The proposed method classified REST, MI-GRASP, and MI-ELBOW with a higher classification accuracy than that achieved using CSP, FBCSP, and band power methods.

The results obtained from the proposed BCI are promising. This method could potentially be used in BCI-driven assistive devices, such as a portable exoskeleton for the arm. For instance, the proposed three-class BCI could increase the controlled number of degrees of freedom for the robotic device designed in our laboratory [31]. This device incorporates mechanical assistance with functional electrical stimulation (FES) either for assistive purposes or for stroke rehabilitation. In a real-life scenario, the participant could imagine “moving her/his elbow” to control the robotic mechanism, which would assist with extending the arm to reach out for an object. The participant could then imagine “grasping with her/his hand” to activate FES, which would assist with grasping the targeted object. For rehabilitation purposes, a similar strategy could be implemented to assist with repetitions of task-specific exercises (e.g. picking up a bean bag and placing it in a different location). Task-specific training after stroke has been shown to produce long-lasting cortical reorganization and has potential for better functional outcomes compared to traditional stroke rehabilitation [56–58].

In future work, we plan to use a higher density EEG system in order to obtain higher resolution activity maps, especially close to the motor cortex. Higher electrode density in this region is likely to improve classification because motor imagery tasks from the same limb activate very similar areas of the motor cortex [20, 21]. We could also investigate the use of ICA or source space analysis with our proposed features, as these processing methods have been reported to improve performance [22, 26]. In addition, the feasibility of classifying rest vs. imaginary grasp and elbow movements within the same limb will be investigated among stroke patients with different levels of impairments. Real-time classification will also be conducted to validate the usefulness of the proposed method in a real-life setting. Future research will focus on reducing the amount of training data required to create a model (in this work, data recorded over one hour, including breaks, for each experiment was used). We also plan to develop a hybrid human machine interface system that combines the proposed EEG-based BCI with EMG placed on the arm. This approach could facilitate improved motor recovery for individuals with a hemiparetic arm resulting from stroke.

## References

[1] Tsui CS, Gan JQ, Roberts SJ. A self-paced brain–computer interface for controlling a robot simulator: an online event labelling paradigm and an extended Kalman filter based algorithm for online training. Med Biol Eng Comput. 2009 3 1; 47(3):257–65. 10.1007/s11517-009-0459-7 19225819

[2] Ang KK, Guan C, Chua KS, Ang BT, Kuah C, Wang C, Phua KS, Chin ZY, Zhang H. Clinical study of neurorehabilitation in stroke using EEG-based motor imagery brain-computer interface with robotic feedback. In: IEEE International Conference in Medicine and Biology. 2010 p. 5549–5552. 10.1109/IEMBS.2010.5626782 21096475

[3] Graimann B, Allison BZ, Pfurtscheller G, editors. Brain-computer interfaces: Revolutionizing human-computer interaction. Springer Science & Business Media; 2010.

[4] He B, Baxter B, Edelman BJ, Cline CC, Wenjing WY. Noninvasive brain-computer interfaces based on sensorimotor rhythms. Proceedings of the IEEE. 2015 6;103(6):907–25. 10.1109/jproc.2015.2407272 PMC8323842 34334804

[5] Dornhege G. Toward brain-computer interfacing. MIT press; 2007.

[6] Pfurtscheller G, Neuper C. Motor imagery and direct brain-computer communication. In: Proceedings of the IEEE. 7; 2001. p. 1123–34.

[7] Herman P, Prasad G, McGinnity TM, Coyle D. Comparative analysis of spectral approaches to feature extraction for EEG-based motor imagery classification. IEEE Trans Neural Syst Rehabil Eng. 2008; 16(4):317–26. 10.1109/TNSRE.2008.926694 18701380

[8] Hamedi M, Salleh SH, Noor AM, Mohammad-Rezazadeh I. Neural network-based three-class motor imagery classification using time-domain features for BCI applications. In: IEEE Region 10 Symposium. 2014. p. 204–207.

[9] Dornhege G, Blankertz B, Curio G, Müller KR. Combining features for BCI. In: Advances in Neural Information Processing Systems. 2002. p. 1115–1122.

[10] McFarland DJ, Anderson CW, Muller K, Schlogl A, Krusienski DJ. BCI meeting 2005-workshop on BCI signal processing: feature extraction and translation. IEEE Trans Neural Syst Rehabil Eng. 2006; 14(2):135 10.1109/TNSRE.2006.875637 16792278

[11] Ramoser H, Müller-Gerking J, Pfurtscheller G. Optimal spatial filtering of single trial EEG during imagined hand movement. IEEE Trans Rehabil Eng. 2000; 8(4):441–447. 11204034 10.1109/86.895946

[12] Ang KK, Chin ZY, Wang C, Guan C, Zhang H. Filter bank common spatial pattern algorithm on BCI competition IV datasets 2a and 2b. Frontiers in neuroscience. 2012 3 29;6:39 10.3389/fnins.2012.00039 22479236 PMC3314883

[13] Ang KK, Chin CY, Zhang H, Guan C. Filter bank common spatial pattern (FBCSP) in brain-computer interface. In: IEEE International Joint Conference on Neural Networks. Hong Kong; 2008. p. 2390–2397.

[14] Geethanjali P, Mohan YK, Sen J. Time domain feature extraction and classification of EEG data for brain computer interface. In: IEEE International Conference on Fuzzy Systems and Knowledge Discovery (FSKD). 2012. p. 1136–1139.

[15] Yong X, Menon C. EEG Classification of Different Imaginary Movements within the Same Limb. PloS one. 2015; 10(4):e0121896 10.1371/journal.pone.0121896 25830611 PMC4382224

[16] Khorshidtalab A, Salami MJ, Hamedi M. Evaluation of time-domain features for motor imagery movements using FCM and SVM. In: IEEE- JCSSE International Joint Conference on Computer Science and Software Engineering. 2012. p. 17–22.

[17] Schlögl A, Lugger K, Pfurtscheller G. Adaptive autoregressive parameters for a brain-computer-interface experiment. In: Engineering in Medicine and Biology Society. 1997. p. 1533–1535. 10.1109/86.712230 9749909

[18] Schlögl A, Neuper C, Pfurtscheller G. Subject specific EEG patterns during motor imaginary. In: IEEE International Conference in Medicine and Biology Society. 1997. p. 1530–1532.

[19] Schlögl A, Lee F, Bischof H, Pfurtscheller G. Characterization of four-class motor imagery EEG data for the BCI-competition. J Neural Eng. 2005; 2(4):L14 10.1088/1741-2560/2/4/L02 16317224

[20] Sanes JN, Donoghue JP, Thangaraj V, Edelman RR, Warach S. Shared neural substrates controlling hand movements in human motor cortex. Science. 1995; 268 (5218):1775–1777. 7792606 10.1126/science.7792606

[21] Plow EB, Arora P, Pline MA, Binenstock MT, Carey JR. Within-limb somatotopy in primary motor cortex—revealed using fMRI. Cortex. 2010; 46(3):310–321. 10.1016/j.cortex.2009.02.024 19446804

[22] Navarro I, Sepulveda F, Hubais B. A comparison of time, frequency and ICA based features and five classifiers for wrist movement classification in EEG signals. In: IEEE EMBS. Shanghai, China; 2005 p. 2118–2115. 10.1109/IEMBS.2005.1616878 17282647

[23] Ghani F, Sultan H, Anwar D, Farooq O, Khan YU. Classification of wrist movements using EEG signals. Journal of Next Generation Information Technology (JNIT). 2013; 4(8):29–39.

[24] Vuckovic A, Sepulveda F. Delta band contribution in cue based single trial classification of real and imaginary wrist movements. Medical Biological Engineering Computing. 2008; 46(6):529–539. 10.1007/s11517-008-0345-8 18418635

[25] Vučković A, Sepulveda F. A two-stage four-class BCI based on imaginary movements of the left and the right wrist. Medical engineering & physics. 2012 9 30;34(7):964–71. 22119365 10.1016/j.medengphy.2011.11.001

[26] Edelman BJ, Baxter B, He B. EEG Source Imaging Enhances the Decoding of Complex Right-Hand Motor Imagery Tasks. IEEE Transactions on Biomedical Engineering. 2016 1;63(1):4–14. 10.1109/TBME.2015.2467312 26276986 PMC4716869

[27] Gu Y, Dremstrup K, Farina D. Single-trial discrimination of type and speed of wrist movements from EEG recordings. Clinical Neurophysiology. 2009 8 31;120(8):1596–600. 10.1016/j.clinph.2009.05.006 19535289

[28] Liao K, Xiao R, Conzalez J, Ding L. Decoding individuals finger movements from one hand using human EEG signals. PLOS ONE. 2014; 9(1):1–12. 10.1371/journal.pone.0085192 PMC3885680 24416360

[29] López-Larraz E, Montesano L, Gil-Agudo Á, Minguez J. Continuous decoding of movement intention of upper limb self-initiated analytic movements from pre-movement EEG correlates. Journal of neuroengineering and rehabilitation. 2014 11 15;11(1):1. 25398273 10.1186/1743-0003-11-153 PMC4247645

[30] Wolpaw JR, Birbaumer N, Heetderks WJ, McFarland DJ, Peckham PH, Schalk G, Donchin E, Quatrano LA, Robinson CJ, Vaughan TM. Brain-computer interface technology: a review of the first international meeting. IEEE Trans Rehabil Eng. 2000; 8(2):164–73. 10896178 10.1109/tre.2000.847807

[31] Looned R, Webb J, Xiao ZG, Menon C. Assisting drinking with an affordable BCI-controlled wearable robot and electrical stimulation: a preliminary investigation. Journal of NeuroEngineering and Rehabilitation. 2014; 11(51):1–13. 24708603 10.1186/1743-0003-11-51 PMC3983865

[32] Electrical Geodesics I. Geodesic Sensor Net Technical Manual. Electrical Geodesics, Inc.; 2007.

[33] Electrical Geodesics I. Net Amps 400 Series Amplifiers;. http://www.egi.com.

[34] Mulert C, Seifert C, Leicht G, Kirsch V, Ertl M, Karch S, Moosmann M, Lutz J, Möller HJ, Hegerl U, Pogarell O. Single-trial coupling of EEG and fMRI reveals the involvement of early anterior cingulate cortex activation in effortful decision making. Neuroimage. 2008 8 1;42(1):158–68. 10.1016/j.neuroimage.2008.04.236 18547820

[35] Tucker DM. Spatial sampling of head electrical fields: the geodesic sensor net. Electroencephalography and clinical neurophysiology. 1993 9 30;87(3):154–63. 7691542 10.1016/0013-4694(93)90121-b

[36] Liu Q, Balsters JH, Baechinger M, van der Groen O, Wenderoth N, Mantini D. Estimating a neutral reference for electroencephalographic recordings: the importance of using a high-density montage and a realistic head model. Journal of neural engineering. 2015 8 26;12(5):056012 10.1088/1741-2560/12/5/056012 26305167 PMC4719184

[37] Pfurtscheller G, da Silva FHL. Event-related EEG/MEG synchronization and desynchronization: basic principles. Clin Neurophysiol. 1999; 110:1842–1857. 10576479 10.1016/s1388-2457(99)00141-8

[38] Dornhege G, Blankertz B, Krauledat M, Losch F, Curio G, Müller KR. Optimizing spatio-temporal filters for improving Brain-Computer Interfacing In: Platt J, editor. Advances in Neural Inf. Proc. Systems (NIPS05). vol. 18 Vancouver, Canada; 2005 p. 315–322.

[39] Wang Y, Gao S, Gao X. Common spatial pattern method for channel selection in motor imagery based Brain-computer Interface. In: Engineering in Medicine and Biology Society, 2005. IEEE-EMBS 2005. 27th Annual International Conference of the IEEE; 2005. p. 5392–5395. 10.1109/IEMBS.2005.1615701 17281471

[40] Khorshidtalab A, Salami MJ, Hamedi M. Robust classification of motor imagery EEG signals using statistical time–domain features. Physiol Meas. 2013; 34(11):1563 10.1088/0967-3334/34/11/1563 24152422

[41] Anderson CW, Sijercic Z. Classification of EEG signals from four subjects during five mental tasks. In: Solving engineering problems with neural networks: proceedings of the conference on engineering applications in neural networks. Turkey; 1996. p. 407–414.

[42] Tseng SY, Chen RC, Chong FC, Kuo TS. Evaluation of parametric methods in EEG signal analysis. Medical engineering & physics. 1995; 17(1):71–8. 7704347 10.1016/1350-4533(95)90380-t

[43] Hamedi M, Salleh SH, Ting CM, Noor AM, Rezazadeh IM. Multiclass self-paced motor imagery temporal features classification using least-square support vector machine. In: IEEE International Conference on Functional Electrical Stimulation Society. 2014. p. 1–5.

[44] Ben-Hur A, Weston J. A user’s guide to support vector machines. Data mining techniques for the life sciences; 2010 p. 223–39. 10.1007/978-1-60327-241-4_13 20221922

[45] Vapnik V. The support vector method of function estimation In: Nonlinear Modeling. Springer US; 1998 p. 55–85.

[46] Hsu CW, Chang CC, Lin CJ. A practical guide to support vector classification.

[47] Bishop CM. Pattern Recognition and Machine Learning. Springer; 2006.

[48] Guger C, Ramoser H, Pfurtscheller G. Real-time EEG analysis with subject-specific spatial patterns for a brain-computer interface (BCI). IEEE Transactions on Rehabilitation Engineering. 2000 12;8(4):447–56. 11204035 10.1109/86.895947

[49] Pfurtscheller G, Neuper C, Schlogl A, Lugger K. Separability of EEG signals recorded during right and left motor imagery using adaptive autoregressive parameters. IEEE transactions on Rehabilitation Engineering. 1998 9;6(3):316–25. 9749909 10.1109/86.712230

[50] Yamawaki N, Wilke C, Liu Z, He B. An enhanced time-frequency-spatial approach for motor imagery classification. IEEE Trans Neural Syst Rehabil Eng. 2006; 14(2):250–4. 10.1109/TNSRE.2006.875567 16792306 PMC1989674

[51] Ang KK, Chin ZY, Zhang H, Guan C. Mutual information-based selection of optimal spatial–temporal patterns for single-trial EEG-based BCIs. Pattern Recognition. 2012; 45(6):2137–44.

[52] Delorme A, Mullen T, Kothe C, Acar ZA, Bigdely-Shamlo N, Vankov A, et al EEGLAB, SIFT, NFT, BCILAB, and ERICA: new tools for advanced EEG processing. Computational Intelligence and Neuroscience. 2011; 2011:1–12. 21687590 10.1155/2011/130714 PMC3114412

[53] Decety J, Peranif D, Jeannerod M, Bettinardif V, Tadary B, Woods R. Mapping motor representations with positron emission. Nature. 1994; 371:13. 10.1038/371600a0 7935791

[54] Rao SM, Binder JR, Bandettini PA, Hammeke TA, Yetkin FZ, Jesmanowicz A, et al Functional magnetic resonance imaging of complex human movements. Neurology. 1993; 43(11):2311–2318. 8232948 10.1212/wnl.43.11.2311

[55] Hollander M, Wolfe DA, Chicken E. Nonparametric Statistical Methods. 3rd ed Wiley; 2013.

[56] Hubbard IJ, Parsons MW, Neilson C, Carey LM. Task-specific training: evidence for and translation to clinical practice. Occupational Therapy International. 2009; 16(3–4):175–189. 10.1002/oti.275 19504501

[57] Rensink M, Schuurmans M, Linderman E, Hafsteinsdottir T. Task-oriented training in rehabilitation after stroke: systematic review. Journal of Advanced Nursing. 2008; 65(4):737–754. 10.1111/j.1365-2648.2008.04925.x 19228241

[58] Classen J, Liepert J, Wise SP, Hallet M, Cohen LG. Rapid plasticity of human cortical movement representation induced by practice. Journal of Neurophysiology. 1998; 79(2):1117–1123. 9463469 10.1152/jn.1998.79.2.1117

